# The London County Council and the Metropolitan Asylums Board

**Published:** 1912-12-14

**Authors:** 


### PROBLEMS IN ADMINISTRATIVE MEDICINE.

(Carefully thought-out Contributions to this Section will be welcome.)

### The London County Council and the Metropolitan Asylums Board.

The recent announcement of prospective co-operation, for purposes of sanatorium benefit under the National Insurance Act, between two great metropolitan administrative bodies, the London County Council and the Metropolitan Asylums Board, is of much interest to institutional workers. At Dartford there happens to be a smallpox hospital belonging to the latter authority, which, though offering accommodation for five hundred patients, has never yet been used in the five or six years now elapsed since its erection. It is a part of the large fever hospital adjoining, which, with the neighbouring mental hospital, makes this Kentish town a somewhat important place in the hospital world.

The provisional arrangement (which has to be sanctioned by the Local Government Board) is for the London County Council to retain the selection of patients and the determining of length of stay, together with certain supervisory privileges; but to employ the Metropolitan Asylums Board to provide accommodation, staff, maintenance, and treatment. This being so, the nature of the existing accommodation seems the first point to be looked to; and, fortunately, it may be said that on the whole it is not unsuitable. In anti-tuberculosis matters we have now got beyond the ideas that ruled ten or even seven years ago. No longer do we find advocates of specially built open-air chapels and similar cost-raising luxuries. The problem of tuberculosis is essentially an economic one; it does not end with medically treating the patient, but involves corollaries like maintaining his dependants and finding him work on discharge which will not throw him back into invalidism. Medical treatment must, therefore, not absorb too large a proportion of available funds after the essentials have been provided for; and about the essentials of phthisis therapeutics experts have now come to an understanding. There must be large window space, but there need be no elaborately glazed lounges: exposure to the sun is discredited since it has been discovered that patients do best in winter.

Rest in bed is absolutely necessary on admission, and generally, one may say, in about one-third of the whole number of cases, taking them all through; but four nurses to every fifty patients will provide the necessary sick attendance. The Dartford accommodation meets these two requirements. As we have previously pointed out, buildings erected for infectious diseases have air space in excess of the normal standard, and with a few easily constructed additional openings, good continuous cross-ventilation could be assured in these twenty-bed pavilions. The nursing quarters are ample, as also those for the domestic staff, both male and female. The building forms a unit quite separate from the rest of the institution, with its own heating, lighting, cooking, and laundry plant, all good of their kind, although not yet used. It should prove of great assistance in the crusade against tuberculosis in London.

The weak point of the scheme arises from the nature of the particular case: smallpox patients are bedridden, and, therefore, easily kept under control, whereas sanatorium consumptives are neither. The separation of the sexes and the enforcement of strict adherence to proper hours and routine will thus be nothing like so easy as in a specially built sanatorium. On this account it would appear highly necessary that the medical superintendent should be one entirely familiar with the difficulties and dangers to be provided against in the case of consumptives. If this be not so, a good many regrettable incidents and scandals can be foreseen. The London County Council rightly acknowledges the experience and high reputation of the Metropolitan Asylums Board in matters administrative. But to manage a sanatorium is quite a different matter from managing a fever hospital or an asylum. Meticulous account keeping, although entirely praiseworthy, is not so much what is wanted as devoted personal attention; that is, if we are to judge by successful examples in the past—namely, the institutions associated with the names of Bodington, Brehmer, Dettweiler, Walther, and Paterson, who were or are all doctors and not stewards.

We think it opportune to make the above remarks for two reasons: First, in the above arrangement it is easy to detect that the dominant note is one of hurry; naturally so, because there will soon be a large number of consumptives claiming some return for the contributions exacted from them in the past. Well, a little delay will bring much less eventual discredit than a wrong start. The Metropolitan Asylums Board numbers among its staff, both lay and medical, many very able persons indeed; but none of them would care to claim much acquaintance with sanatorium work. If, as is rumoured, the medical and nursing staff is to be appointed almost immediately, there would seem small opportunity of getting the best available *personnel*. This becomes more important because of the second point to be borne in mind—namely, that although it is expressly stated that the idea is for sanatorium treatment at the Dartford institution to be only temporary, yet it is also implied that the same management would prevail were the *venue* changed. A great administrative body like the Metropolitan Asylums Board has a right to be proud of its work, both past and present, but it will doubtless bear well in mind the fact that it is undertaking work to which it is entirely new and strange, and proceed at first with caution and tentatively.

